# Whole genome sequence analysis of equid gammaherpesvirus -2 field isolates reveals high levels of genomic diversity and recombination

**DOI:** 10.1186/s12864-022-08789-x

**Published:** 2022-08-30

**Authors:** Adepeju E. Onasanya, Charles El-Hage, Andrés Diaz-Méndez, Paola K. Vaz, Alistair R. Legione, Glenn F. Browning, Joanne M. Devlin, Carol A. Hartley

**Affiliations:** 1 Melbourne Veterinary School, Faculty of Veterinary and Agricultural Sciences, The Asia-Pacific Centre for Animal Health, The University of Melbourne, Parkville, VIC 3010 Australia; 2 Centre for Equine Infectious Diseases, Faculty of Veterinary and Agricultural Sciences, The University of Melbourne, Parkville, VIC 3010 Australia

**Keywords:** Equid gammaherpesvirus 2, EHV2, Recombination, Genome sequence, Diversity

## Abstract

**Background:**

Equid gammaherpesvirus 2 (EHV2) is a gammaherpesvirus with a widespread distribution in horse populations globally. Although its pathogenic significance can be unclear in most cases of infection, EHV2 infection can cause upper respiratory tract disease in foals. Co-infection of different strains of EHV2 in an individual horse is common. Small regions of the EHV2 genome have shown considerable genetic heterogeneity. This could suggest genomic recombination between different strains of EHV2, similar to the extensive recombination networks that have been demonstrated for some alphaherpesviruses. This study examined natural recombination and genome diversity of EHV2 field isolates.

**Results:**

Whole genome sequencing analysis of 18 EHV2 isolates, along with analysis of two publicly available EHV2 genomes, revealed variation in genomes sizes (from 173.7 to 184.8 kbp), guanine plus cytosine content (from 56.7 to 57.8%) and the size of the terminal repeat regions (from 17,196 to 17,551 bp). The nucleotide sequence identity between the genomes ranged from 86.2 to 99.7%. The estimated average inter-strain nucleotide diversity between the 20 EHV2 genomes was 2.9%. Individual gene sequences showed varying levels of nucleotide diversity and ranged between 0 and 38.1%. The ratio of nonsynonymous substitutions, Ka, to synonymous substitutions, Ks, (Ka/Ks) suggests that over 50% of EHV2 genes are undergoing diversifying selection. Recombination analyses of the 20 EHV2 genome sequences using the recombination detection program (RDP4) and SplitsTree revealed evidence of viral recombination.

**Conclusions:**

Analysis of the 18 new EHV2 genomes alongside the 2 previously sequenced genomes revealed a high degree of genetic diversity and extensive recombination networks. Herpesvirus genome diversification and virus evolution can be driven by recombination, and our findings are consistent with recombination being a key mechanism by which EHV2 genomes may vary and evolve.

**Supplementary Information:**

The online version contains supplementary material available at 10.1186/s12864-022-08789-x.

## Background

Equid gammaherpesvirus 2 (EHV2) is the type species in the genus *Percavirus* of the subfamily *Gammaherpesvirinae* in the order *Herpesvirales* [21]. The widespread presence of EHV2 in horses worldwide is an indication of its evolutionary success [35]. The ubiquitous presence of EHV2 in horses has made definitive association with clinical disease challenging since it is detected in horses showing clinical signs of disease and in healthy horses [19, 26, 48, 58, 91], including in Icelandic horses which have been isolated from direct contact with other horse populations for over 1000 years [92]. A range of clinical signs have been associated with EHV2 infection, including mild to severe upper respiratory tract disease, and keratoconjunctivitis [5, 9, 44, 58, 99]. EHV2 has been detected and isolated from a variety of tissues and samples including the trachea, lymph nodes, lung, spleen, kidney, gastric mucosal epithelium, bronchoalveolar lavage fluid, as well as nasal and ocular swabs of both healthy horses and those showing different signs of clinical disease [28, 29, 65, 92]. Herpesviruses establish lifelong latent infection within the host at varying sites [1, 71]. B lymphocytes are the major site of latency for EHV2 infection [23, 55].

The EHV2 genome contains 57.7% guanine and cytosine (G + C) bases and encodes 79 open reading frames (ORFs) which are arranged into a unique region (UR) with internal repeats (IR1- IR1L, IR1R, and IR2- IR2L, IR2R), flanked by direct terminal repeats (TRs) at both ends. The genome sizes of EHV2 strains 86–67 and G9/92 (GenBank accession numbers NC_001650 and KM924294, respectively) are 184,439 and 186,110 bp, with direct TRs of 17,553 and 18,332 bp, respectively [13, 14, 87, 102]. Most of the available EHV2 sequences are partial genome sequences, with the most frequently studied genomic regions including glycoprotein B (gB), glycoprotein H (gH), DNA polymerase and terminase genes [13, 14, 36, 58, 75, 91]. High levels of genomic variability have been detected in the gB and gH genes [83, 91] and in ORFs E1, 74 and E6 [75, 87]. The latter three are homologues of cellular seven-transmembrane receptors (7TMR), which in other gammaherpesviruses can act as functional G protein-coupled receptors (GPCR) [16, 22, 45, 60].

Co-infection of different EHV2 strains within the same horse has been reported in previous studies [9, 11, 12, 14, 15, 75, 91]. Co-infection is a fundamental requirement for viral recombination, which is one mechanism by which herpesviruses may achieve genome variation and diversification [10, 50, 57, 66, 95]. Recombination in gammaherpesviruses has not been widely studied except in Epstein Barr virus (EBV, human gammaherpesvirus-4) [8].

This study aimed to investigate EHV2 genome variation and recombination by the determination and analysis of the full genome sequences of historical (archived) and contemporary EHV2 isolates from Australian horses.

## Results

### Culture of contemporary EHV2 isolates from peripheral blood mononuclear cells (PBMCs)

Contemporary EHV2 isolates were collected by co-culture of peripheral blood mononuclear cells (PBMCs) originating from 5 different horses, with equine fetal kidney (EFK) cell monolayers after 5 to 21 days of incubation. Of 149 plaques, most were identified as either EHV2 or equid gammaherpesvirus 5 (EHV5) by polymerase chain reaction (PCR) (Table 1). In total, more plaques tested positive to only EHV2 (*n* = 58/149, 39%) compared to plaques positive for only EHV5 (*n* = 26/149, 17%) (Table 1). To ensure distinct EHV2 isolates were selected for genome analysis, plaque purified isolates from the PBMC co-cultures were initially characterised by qPCR-high resolution melt (qPCR-HRM) curve analysis of the gB gene and then a subset of the 58 EHV2-positive isolates (*n* = 8) with distinct melt profiles were selected for sequencing (Table 1) along with a further 10 EHV2 historical isolates (Table 2).

**Table 1:** Equid gammaherpesvirus detection in PBMC-EFK co-cultures by qPCR-HRM targeting the glycoprotein B ORF

Horse no	Host	Single virus positive		EHV2 & 5 positive ^b^	EHV2 & 5 negative ^c^	Isolates sequenced ^d^	HRM Tm °C ^e^
		**EHV2 **^**b**^	**EHV5 **^**b**^				
1	4 yr old TB ^a^	8/13 (61%)	1/13 (8%)	4/13 (31%)	0/31	H1-3–2018	86.8
2	17 yr old TB ^a^	14/67 (21%)	17/67 (25%)	34/67 (51%)	2/67 (3%)	H2-80–2018	89.0
3	23 yr old pony	8/16 (50%)	3/16 (19%)	2/16 (12%)	3/16 (19%)	H3-25–2018	89.9
4	34 yr old pony	8/22 (36%)	5/22 (23%)	2/22 (9%)	7/22 (32%)	H4-147–2018	87.0
						H4-18–2018	88.5
5	Retired TB ^a^	20/31 (65%)	0/31 (0%)	10/31 (32%)	1/31 (3%)	H5-91–2018	88.3
						H5-57–2018	85.0
						H5-60–2018	89.2
*Total*		*58/149 (39%)*	*26/149 (17%)*	*52/149 (35%)*	*13/149 (9%)*		

^a^ TB = Thoroughbred

^b^ Number (percentage) of viral plaques positive for EHV2 only, EHV5 only, or both viruses (dual positive)

^c^ Number (percentage) of viral plaques where neither EHV2 nor EHV5 were detected

^d^ H(1–5) denotes the horse number from which isolates were recovered

^e^ HRM Tm = high resolution melting temperature

**Table 2:** Details of archived and contemporary Australian EHV2 isolates used in this study

EHV2 Virus isolate ID	Year	Type ^a^/Similarity	Location ^b^	Source	References	Virus passage ^c^	GenBank accession number	SRR accession number
1–141-67	1967	1–141-67	Parkville	Nasal	Turner et al.,1970	23	MW322569	SRR17631889
2–16-67	1967	86–67	Parkville	Nasal	Turner et al.,1970	21	MW322570	SRR17631888
ER34-67	1967	1–141-67	Bundoora	Nasal	Turner & Studdert, 1970	14	MW322583	SRR17631879
1039–94	1994	1–141-67	Euroa	Nasal	[36]	8	MW322581	SRR17631878
691–82	1982	86–67	Bacchus Marsh	Pharyngeal	Browning & Studdert, 1974	9	MW322580	SRR17631877
Fin60-72	1972	86–67	Laverton	Nasal	Wilks & Studdert, 1974	9	MW322584	SRR17631876
Fin150-72	1972	86–67	Laverton	Nasal	Wilks & Studdert, 1974	9	MW322585	SRR17631875
Fin180-72	1972	86–67	Laverton	Nasal	Wilks & Studdert, 1974	9	MW322586	SRR17631874
9951E-69	1969	1–141-67	Frankston	Eye	Browning et al.,1988b	10	MW322582	SRR17631873
157IFeye-69	1969	86–67	Frankston	Eye	Studdert,1971	11	MW322579	SRR17631872
H1-3–2018	2018	86–67	Wallington	PBMC	Present study	6	MW322571	SRR17631887
H2-25–2018	2018	86–67	Wallington	PBMC	Present study	6	MW322573	SRR17631886
H3-80–2018	2018	1–141-67	Wallington	PBMC	Present study	6	MW322576	SRR17631885
H4-18–2018	2018	86–67	Wallington	PBMC	Present study	6	MW322572	SRR17631884
H5-91–2018	2018	86–67	Wallington	PBMC	Present study	6	MW322577	SRR17631883
H5-57–2018	2018	86–67	Wallington	PBMC	Present study	6	MW322574	SRR17631882
H5-60–2018	2018	86–67	Wallington	PBMC	Present study	6	MW322575	SRR17631881
H4-147–2018	2018	86–67	Wallington	PBMC	Present study	6	MW322578	SRR17631880

^a^ Type based on gB (site 1) sequence [36]

^b^ All locations are suburbs or towns in Victoria, Australia

^c^ Virus passage number (in EFKs) at which the viruses were used in this present study

### Complete genome sequencing of 18 Australian EHV2 field isolates

**Fig. 1:**
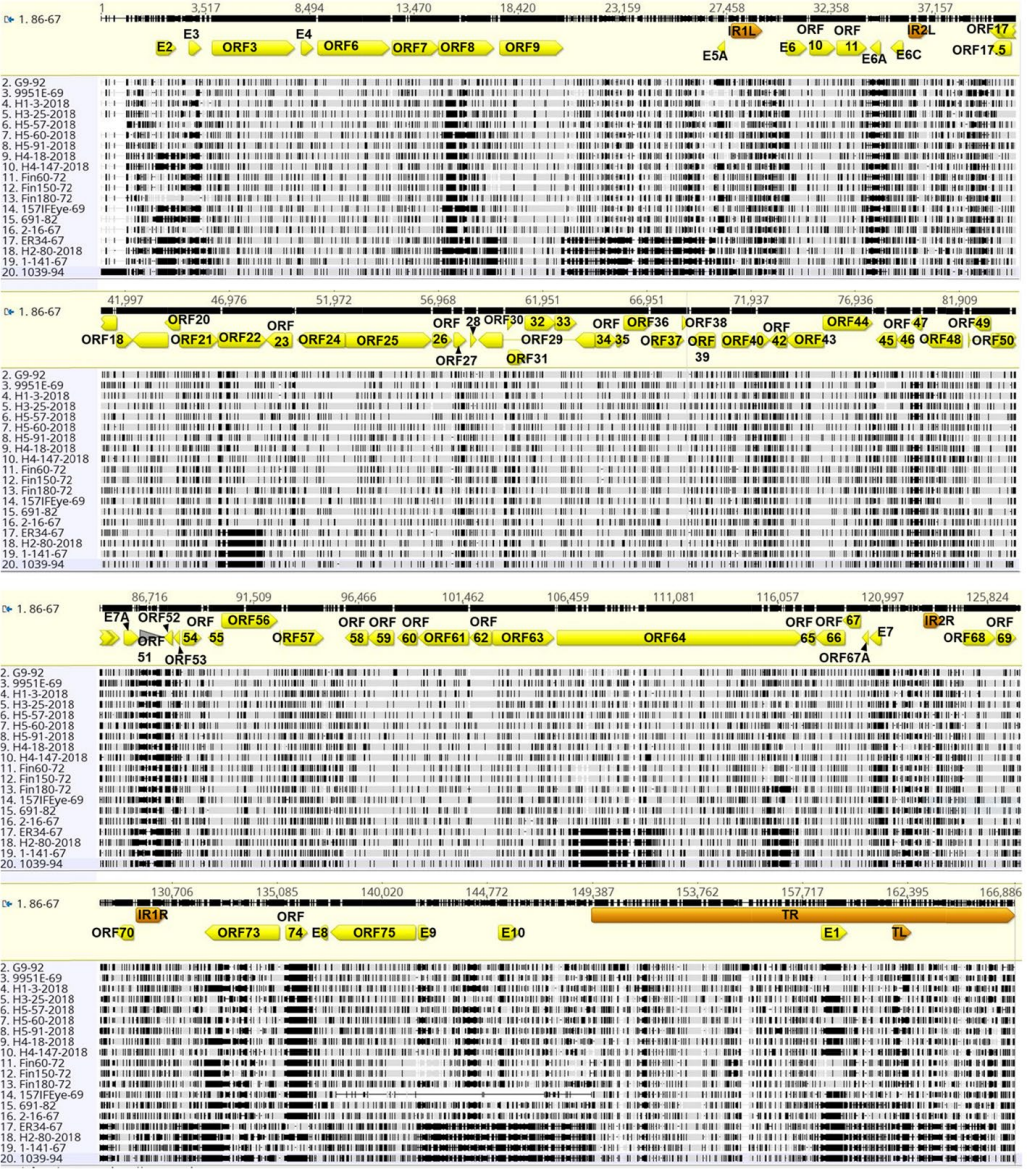
Nucleotide sequence alignment of 20 EHV2 genome sequences, including strains EHV2 86–67 (GenBank accession NC_001560) and G9-92/92 (GenBank accession KM924294)

Assembly of the genome was done with only one terminal repeat since they are direct repeats leaving the reference genome size at 166,886 bp instead of 184,439 bp for the full genome. Genome sequences of the 18 EHV2 isolates were assembled by mapping to the reference genome EHV2 86–67 (all isolates) and by de novo assembly (two isolates: Fin60-72 and 157IFEye-69). Comparison of the two genome sequences assembled by both reference mapping and de novo assembly methods showed that most disagreements between the two methods occurred in highly variable regions containing insertions/deletions (INDELs) and in repeat rich regions, particularly at the terminal repeats (TR). The genomes produced by the two methods of assembly (de novo and reference assisted) shared 89.7% pairwise identity in the TR region of Fin60-72 and 91.3% for 157IFEye-69. By comparison, their unique regions showed 97.9% identity for Fin60-72 identify for de novo assembly and 97.3% identity for 157IFEye-69.for reference assisted assembly. The internal repeat regions IR1L, IR1R, IR2L, and IR2R assembled by de novo and reference-assisted methods had 99%, 100%, 88.4% (due to 96 bp gap in de novo assembly) and 98.9% identities respectively for 157IFEye-69, and 100%, 100%, 98.1% and 98.5% for Fin60-72, respectively. Alignments of the genomes produced by the two methods of assembly (see Supplementary Figs. 1 and 2, Additional file 1). The genome length (including only 1 TR) produced using each method was comparable for both isolates, where the respective map to reference and de novo coverage was 166,763 bases compared to 166,709 bases, respectively, for Fin60-72, and 156,230 bases compared to 156,141 bases, respectively, for 157IFEye-69. Regions of difference between the two methods containing INDELs in some genes (ORFs 29, 34 and 48) were amplified by PCR in order to ascertain their sizes. The size of the PCR amplicons reflected the expected sizes of 400, 942 and 671 bp respectively) consistent with the map to reference method of genome assembly, rather than the de novo assembly method, which predicted sizes of 613, 465, and 361 bp respectively. The map to reference method was therefore used for determining the full genome sequences of all 18 Australian isolates of EHV2 in this study. Annotation of ORFs was compared to EHV2 strains 86–67 and G9/92 as references to identify discrepancies and validate the annotation method. The alignment of the 20 whole genome sequences (18 sequences from this study, along with the sequences of strains 86–67 and G9/92) is shown in Fig. 1.

Alignment of the complete genome sequences was performed using MAFFT. The prototype strain EHV2 86–67 with the first terminal repeat (TR) removed was used as the reference sequence. Vertical black lines indicate SNPs compared to the reference and dashes indicate sequence gaps.

### Nucleotide diversity of EHV2 whole genome sequences

The average size of the complete genomes containing both TRs is 183,470 bp and ranged from 173,753 bp (157-IFEye-69) to 184,828 bp (1039–94), where the genome of isolate 157-IFEye-69 had a large deletion resulting in the absence of ORFs 75, E9 and E10. A summary of sequence and assembly metrics is provided (see Supplementary Table 1, Additional file 2). The previously published sequence of strain 86–67 encodes a truncated ORF51 (homologue of EBV gp350) while the strain G9/92 encodes the full-length protein. Three other isolates in this study also contained mutations in ORF51. These, along with other genes containing mutations that disrupt ORFs (INDELs indivisible by three or deletions resulting in a frameshift) were predicted to result in a truncated protein being expressed as summarised in Table 3.

**Table 3:** Genes containing ORF-disrupting mutations

Gene	Type of mutation	Isolate	ORF length (bp) ^b^	Predicted protein (aa)
E6A	Deletion ^a^	86–67	348	116 (FL ^d^)
		691–82	195^c^	64
		Fin180-72	231^c^	76
ORF7	Deletion ^a^	86–67	2187	729 (FL ^d^)
		1–141-67	2185	230
ORF51	Deletion ^a^	G9-92	837	279 (FL ^d^)
		147–2018	826	33
		86–67	831	97
		1–141-67	288	95
		2–16-67	723	62
ORF64	Deletion ^a^	86–67	11,268	3755 (FL ^d^)
		80–2018	11,281	899
		1039–94	11,254	899
		ER34-67	11,248	1498
		1–141-67	11,197	948
ORF74	Deletion ^a^	86–67	993	330 (FL ^d^)
		3–2018	1000	83
ORF75	Deletion ^a^	86–67	4038	1345 (FL ^d^)
		157IFEye-69	840	178
E9	Complete gene deletion	157IFEye-69		
E10	Complete gene deletion	157IFEye-69		

^a^ deletion (with some indivisible by 3) resulting in a premature stop codon

^b^ open reading frame

^c^ frame shift resulting in premature stop codon and shortened ORF

^d^ expected number of amino acids in full length (FL) product as predicted in already published sequences, reference strain 86–67 or strain G9/92

The percentage nucleotide identity between the 20 genome sequences revealed a high level of genetic diversity amongst EHV2 strains, where nucleotide identities ranged from 86.2 to 99.7%. A nucleotide identity matrix is provided (see Supplementary Table 2, Additional file 2).

**Fig. 2:**
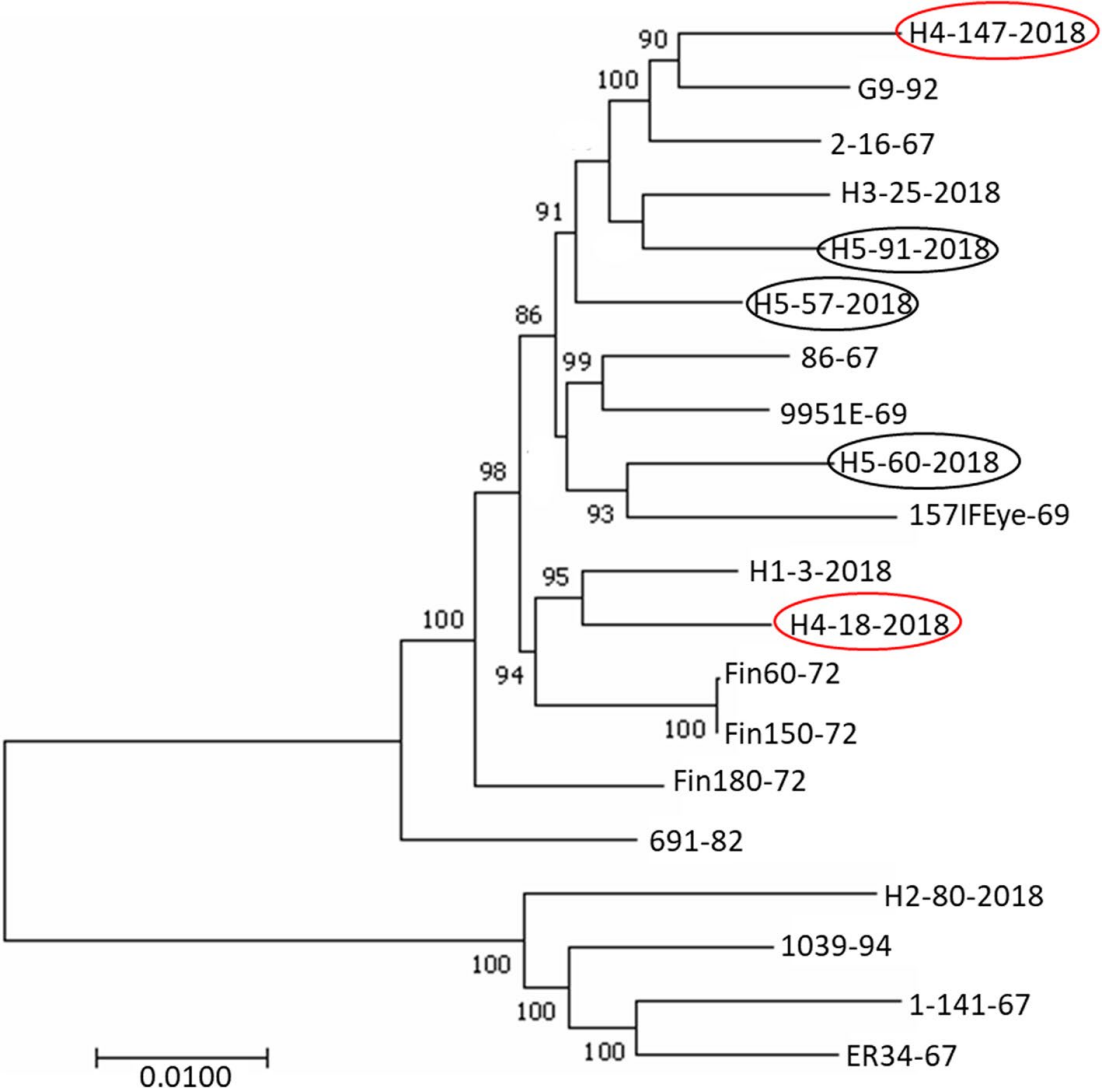
Maximum likelihood phylogenetic tree of the 20 complete genomes of EHV2 isolates. Two distinct groups are apparent (86–67-like and 1–141/67- like). Phylogenetic analysis was inferred by using the Maximum likelihood method based on the General Time Reversible model (GTR) using 1000 bootstrap replicates. The percentage (> 80%) of trees in which the associated taxa clustered together is shown next to branches. The trees were initially built by applying BioNJ method to a matrix of pairwise distances estimated using the Maximum Composite Likelihood (MCL) approach. The tree is drawn to scale, with branch lengths measured in the number of substitutions per site as indicated on the scale bar and bootstrap values are shown. Isolates in oval (red or black) indicate those recovered from the same horse

The phylogenetic analysis revealed EHV2 isolates grouped into 2 distinct clusters, with most of the isolates clustering with strain 86–67 (including G9/92 isolated in the UK), whereas only 1 contemporary and 3 archived isolates clustered with 1–141/67 (Fig. 2).

Genome sequence polymorphism was interrogated by DnaSP analysis [72, 77]. Alignment of the 20 whole genomes contained 175,141 nucleotide sites. Of these, 149,886 sites contained no gaps and 9.1% (15,879) of these sites were polymorphic (S). The average number of nucleotide differences (k) between any two genomes was 4323. Estimated inter-strain nucleotide diversity (π) was 0.029 which represents 2.9% of the analysed sequence sites across the full genome. We analysed the π values of other selected viruses using publicly available complete genome sequences. For equine alphaherpesvirus 4 (EHV4, *n* = 14) and equine alphaherpesvirus 1 (EHV1, *n* = 22) strains published by Vaz et al. [95], π was much lower, at 0.0014 and 0.0011, respectively. The inter-strain diversity of EHV2 is more comparable to that of the highly variable betaherpesvirus human cytomegalovirus (HCMV, *n* = 124, π = 0.021) [77] than that of the gammaherpesvirus EBV (*n* = 60, π = 0.0079) [64].

### Analysis of the diversity and divergence of EHV2 genes

**Fig. 3:**
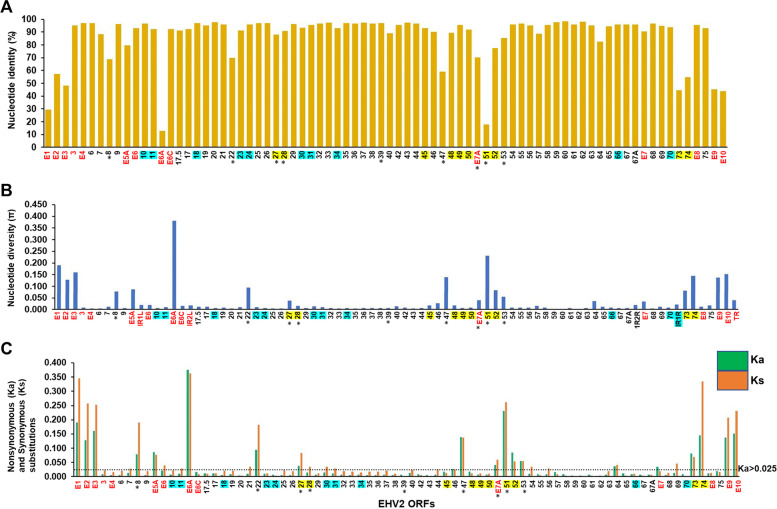
Analysis of the genetic divergence and diversity of all 78 EHV2 genes using the 20 aligned EHV2 genome sequences.** A** Mean % nucleotide identity. **B** Mean nucleotide diversity (π) values which represents the average number of nucleotide substitutions per site (excluding gaps). **C** Mean Ka values (representing nonsynonymous substitutions—green bars) and Ks values (representing synonymous substitutions—orange bars). Ka values < 0.002 are considered most conserved, whilst values > 0.025 are considered most diverged [77]. In panel B, the equid gammaherpesvirus (EGHV) specific genes are in red font, genes specific to both Betaherpesviruses and Gammaherpesviruses and are shaded blue, and Gammaherpesviruses specific genes are shaded yellow. Unshaded genes are conserved genes in all herpesviruses. Glycoproteins are marked with an asterisk (*). IR = internal repeats, and TR = terminal repeats

The percentage nucleotide identity amongst the 20 EHV2 isolates for each gene was evaluated from the alignment of each gene sequence (Fig. 3a). The average nucleotide diversity (π) for individual gene sequences was determined for all pairwise comparisons of each gene from the 20 isolates (Fig. 3b). Genes with low nucleotide diversity values (Ka < 0.002) in EHV2 were mostly involved in DNA-processing (ORFs 6, 9, 18, 25, 26, 44 and 54), while genes with high diversity values (Ka > 0.025) in EHV2 encoded structural proteins such as glycoproteins (gB, gH, gL, gp48, gM) and tegument proteins (ORFs 52 and 64). Other genes showing high diversity includes ORFs 73, 74 and 51. Nonsynonymous substitutions per nonsynonymous site (Ka) and synonymous substitutions per synonymous site (Ks) were determined for each of the 78 EHV2 genes (Fig. 3c). Nine EHV2 genes (12%) had Ka values < 0.002 (conserved) (Table 4), while 19 EHV2 genes (24%) had a value > 0.025 (divergent) (Fig. 3c, Table 5) [77].

**Table 4:** Highly conserved EHV2 genes (Ka values ≤ 0.002)

ORF	Ka ^a^	Gene Family	Gene Conservation ^b^	Gene product	Functions
6	0.001	DNA binding protein	Core	ssDNA binding protein	DNA replication and possibly involved in gene regulation
9	0.001	DNA polymerase type B	Core	DNA polymerase	DNA replication
10	0.002	Deoxyuridine triphosphatase-related protein (DURP)	Beta/Gamma	Protein G10	Unknown
18	0.001	UL79	Beta/Gamma	Protein UL79	Promote accumulation of late transcript
25	0.000	Major capsid protein	Core	Major capsid protein	Capsid morphogenesis
26	0.001	TRX2 protein	Core	Capsid triplex subunit 2	Capsid morphogenesis
33	0.001	UL16	Core	Tegument protein UL16	Possibly involved in virion morphogenesis
35	0.001	UL14	Core	Tegument protein UL14	Virion morphogenesis
36	0.002	Conserved herpesvirus protein kinase (CHPK)	Core	Tegument serine/threonine protein kinase	Protein phosphorylation
44	0.001	Helicase	Core	Helicase-primase helicase subunit	DNA replication
54	0.001	DURP	Core	Deoxyuridine triphosphatase	Nucleotide metabolism

^a^ Ka = nonsynonymous substitutions per nonsynonymous site

^b^ Gene conservation over different herpesvirus subfamilies; Core = conserved in all herpesviruses, Beta/Gamma = conserved in betaherpesvirinae and gammaherpesvirinae subfamilies

**Table 5:** Highly divergent EHV2 genes (Ka values > 0.025)

ORF	Ka ^a^	Gene family ^b^	Gene conservation ^c^	Gene product	Functions
E1	0.139	GPCR 1	EHV2	E1	Intracellular signalling (7TMR domains)
E2	0.089	NA	EHV2	E2	Signalling peptide (Ig domain)
E3	0.130	Complement Control Protein (CCP)	EHV2	E3	Membrane protein
8	0.042	Glycoprotein B	Core	gB	Cell entry and cell to cell spread
E5A	0.090	NA	EHV2	E5A	Unknown
E6A	0.285	E6A	EHV2	E6A	Contain signal peptide
22	0.067	Glycoprotein H	Core	gH	Cell entry and cell to cell spread
46	0.027	Uracil DNA Glycolase (UNG)	Core	uracil-DNA glycosylase	DNA repair
47	0.138	Phage_glycop_gL	Core	gL	Tegument
E7A	0.033	NA	Gamma	g42	Cell entry, binds MHC-II (C-type lectin-like domain)
51	0.208	Epstein-Barr GP350	Gamma	gp350	Cell attachment
52	0.093	BLRF2	Core	virion protein G52	
53	0.052	Glycoprotein N	Core	gN	Virion morphogenesis and membrane fusion
64	0.036	large tegument protein	Core	large tegument protein	Capsid transport
E7	0.041	IL-10	EHV2	IL-10	Immune regulation
73	0.086	NA	Gamma	nuclear antigen LANA-1	Involved in latency
74	0.084	GPCR 1	Beta/Gamma	Membrane protein G74	Intracellular signalling (7TMR domains)
E9	0.109	NA	EHV2	Membrane protein E9	
E10	0.143	CARD	EHV2	apoptosis regulator E10	Involved in apoptosis

^**a**^ Ka = nonsynonymous substitutions per nonsynonymous site

^b^ NA = not assigned to a gene family

^c^ Gene conservation over different herpesvirus subfamilies: Core = conserved in all herpesviruses, Gamma = conserved in all members of the subfamily gammaherpesvirinae, Beta/Gamma = conserved in all members of the betaherpesvirinae and gammaherpesvirinae subfamilies, EHV2 = EHV-2-specific gene

The EHV2 core genome contains 13 genes that are unique to the EGHVs (EHV2 and EHV5). These genes, annotated with the ‘E’ prefix are located towards both genomic termini, or close to the internal repeat regions and mostly (9 of 13) displayed high diversity (Ka values > 0.025 and π values 0.035 – 0.381) (Fig. 3, Table 5, and Supplementary Table 3, Additional file 2).

The Ka/Ks ratio is used as a measure of the selection pressure acting on a gene [98]. A ratio close to zero indicates strong negative/purifying selection, a ratio close to 1 indicates neutral selection or genetic drift, while a ratio higher than 1 indicates positive/diversifying selection. Almost half of the EHV2 genes (47%) had a Ka/Ks ratio < 0.5, 17% had a ratio between 0.5 and 1, and 36% had a ratio > 1 (see Supplementary Table 3, Additional file 2). Only a weak correlation (Spearman’s rank correlation analysis, *ρ* = 0.212, *P* = 0.06) was observed between selection pressure (Ka/Ks ratio) and nucleotide diversity (π) of EHV2 genes. High Ka/Ks ratios do not directly translate to a high diversity and vice versa.

The gB, gH and DNA polymerase genes are commonly investigated in EHV2 studies and thus additional analyses were performed for gB and gH genes that incorporated other publicly available sequences from other EHV2 isolates (see Supplementary Table 4, Additional file 2 for details of other sequences). Of these 3 genes, the highest nucleotide identities were observed for the DNA polymerase gene, which also had the lowest Ka value (0.001) (Table 4). All three genes had Ka/Ks ratios < 0.5 suggesting negative selection (see Supplementary Table 3, Additional file 2).

### Amino acid sequence analysis of EHV2 gB sites

**Fig. 4:**
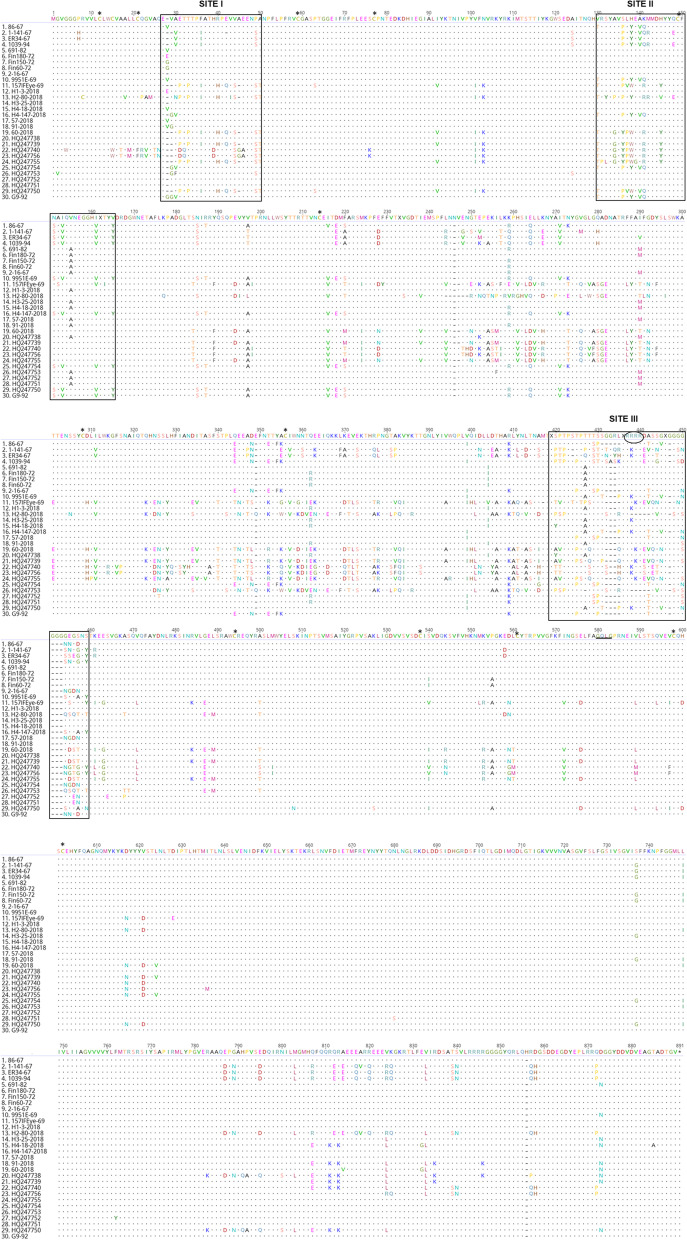
Amino acid alignment of 30 complete EHV2 glycoprotein B gene sequences. Australian isolates (numbers 1 – 19, where 1–11 are historical and 12–19 contemporary), Icelandic isolates (numbers 20—22), other international isolates (numbers 23 – 30) [91, 102]. Site I (aa 28—50), Site II (aa 130—165), Site III (aa 419—459) and aa 230 and 270 labelled as previously described antigenic or variable sites [11, 36]. 13 cysteine residues (asterisk marked). Endoproteolytic cleavage site (aa 437—440, black oval within site III). GQLG sequence (aa 580—583, underlined)

The amino acid sequence of the gB from 30 EHV2 strains (including 18 determined in this study and 12 sourced from GenBank, see Supplementary Table 4, Additional file 2) showed conservation of both the proposed endoproteolytic furin cleavage site R-X-K/R-R at residues 437–440 and the GQLG sequence at residues 580–591 reported to be conserved in all herpesviruses examined. The 13 cysteine residues are also conserved in the 30 gB sequences including synonymous substitution observed in two foreign isolates [34, 36, 75, 81]. Antigenic sites of EHV2 gB had previously been characterised based on variability of 4 strains (86–67, 2–141-67, 5FN and T-2) and their reactivity to a panel of monoclonal antibodies [36]. Variability amongst the 30 gB aa sequences within the antigenic site I (aa 27–51), show 18 isolates were 86–67-like and shared between 89.47% to 100% aa identity at this site. The antigenic sites designated II and III (Fig. 4) are further confirmed as regions of high variability amongst EHV2 strains and the 30 strains shared 55–100% and 31–100% aa identities at these sites, respectively. Figure 4 also shows regions of amino acid variation beyond those previously identified antigenic sites.

### Phylogenetic analysis of EHV2 GPCR gene family

**Fig. 5:**
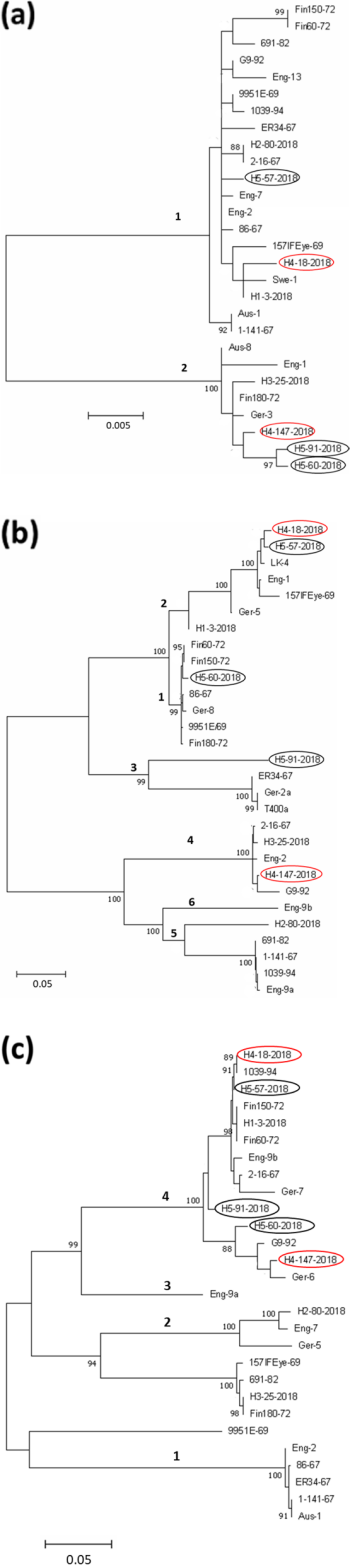
Maximum- likelihood phylogenetic trees for complete nucleotide sequences of (a) E6, (b) E1, and (c) 74 genes for a panel of EHV2 Australian isolates and representatives of previously reported EHV2 genogroups for each gene [75]. The genogroup designation from a previous study are shown by the numbers [75]. Phylogenetic analysis was inferred by using the Maximum likelihood method based on the General Time Reversible model (GTR) using 1000 bootstrap replicates. The percentage (> 80%) of trees in which the associated taxa clustered together is shown next to branches. The trees were initially built by applying BioNJ method to a matrix of pairwise distances estimated using Maximum Composite Likelihood (MCL) approach. The tree is drawn to scale, with branch lengths measured in the number of substitutions per site as indicated on the scale bar. Isolates in oval (red or black) indicate those recovered from the same horse

EHV2 encodes 3 GPCR genes (ORF74, E1, and E6) which share some similarities to cellular chemokine receptors [87]. The nucleotide sequences of E6, ORF74 and E1 from this study were aligned with sequences (see Supplementary Table 4, Additional file 2) representing previously identified genogroups [75]. The genogroups identified by phylogenetic analysis in the current study were consistent with previous findings [75] except for ORF74. E6 was the least variable gene and divided the isolates into 2 clear genogroups (Fig. 5A), while E1 generally grouped in the 6 existing genogroups (Fig. 5B). ORF74 grouped similarly to the Sharp study [75] although several additional sequences identified in the current study might form a new genogroup (Fig. 5C). Consistent with this, both ORF74 and E1 are amongst the most divergent genes in EHV2, Ka > 0.025 (Table 5) with Ka/Ks ratios of 0.25 and 0.4, respectively, indicating they are under a negative/purifying selection (see Supplementary Table 3, Additional file 2). It was observed that the EHV2 viruses recovered from the same horse did not share similar genogroups in all the GPCR genes (E6, E1, and ORF74) (Fig. 5).

### Genome diversity of EHV2 recovered from individual horses

Consistent with their PCR-HRM results, the EHV2 isolates recovered from the same horses at the same time were not identical. Isolates recovered from Horse 4 (147/2018 and 18/2018) shared 97% identity, while isolates from Horse 5 (91/2018, 57/2018 and 60/2018) shared an average of 95% identity across their genomes, including the genome termini. The similarity plot between isolates from the two horses show a similar trend of variability along the genome, highest at the termini (see Supplementary Figs. 3 and 4, Additional file 1).

### Recombination analysis of EHV2 genome

**Fig. 6:**
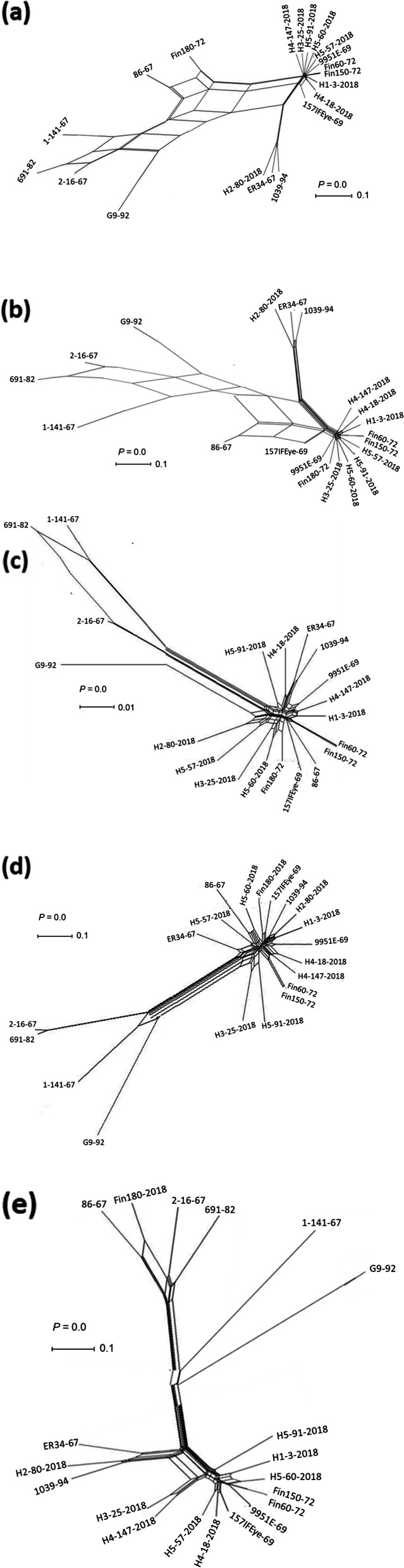
Recombination network trees generated from EHV2 nucleotide alignments (excluding tandem repeats) of 20 EHV2 isolates using SplitsTree4**.** **A** Complete genome sequences **B** unique region **C** internal repeat 1 **D** internal repeat 2 and **E** terminal repeat region. The multiple reticulate networks indicate recombination events between the different isolates. The bar indicates the rate of evolution in sequence substitutions per site. The Phi test for detecting recombination as implemented in SplitsTree4 was significant (*P* < 0.05) for the complete genome and other genome regions analysed

**Fig. 7:**
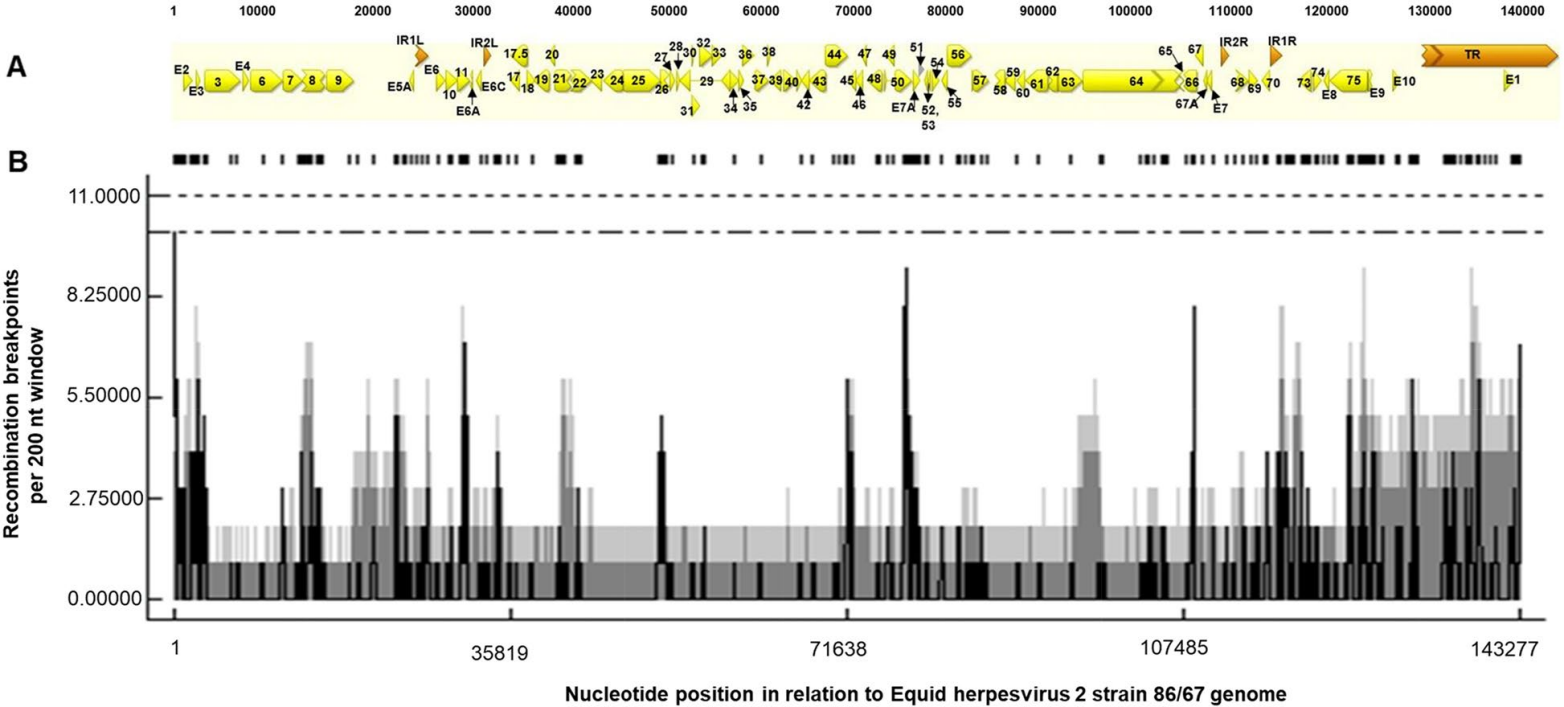
Detection of recombination breakpoints in the alignment of 20 EHV2 genomes.** A **Schematic representation of EHV2 genome annotation including 78 ORFs, CDS (yellow), repeats regions (the orange forward facing arrows- internal repeats IR smaller arrows within, and terminal repeats TR at the far right). The labels indicate the positions of the ORFs along the genome length. **B** Recombination breakpoints detected per 200 nucleotide (nt) window. The vertical lines represent recombination breakpoints per 200 nt window in each analysed sequence, as detected with 95% confidence (grey) or 99% confidence in (black). Horizontal lines indicate the limits for global hot spot detection, indicated at 95% confidence (**) or 99% confidence (+ +)

The 20 aligned EHV2 genome sequences were examined for evidence of recombination across the complete (excluding the left TR) and unique genome regions, as well as repeat regions, IR (1 and 2), and TR using SplitsTree4 [38]. The reticulate networks and pair-wise homoplasy index (Phi) test detected significant recombination between the 20 EHV2 strains in all the genomic regions analysed (Fig. 6). Reticulate phylogenetic recombination networks were also generated for some genes (gB, gH and the 3 GPCRs) (see Supplementary Fig. 5, Additional file 1). The RDP4 program was used to detect recombination events and recombination breakpoints. Evidence of multiple recombination events was detected (Fig. 6 and Supplementary Table 5, Additional file 2) and recombination breakpoints were widespread through the length of the genome (Fig. 7). Overall, 155 recombination events were reported, representing those detected by 3 programs or more. Of these 105 were detected by 5 or more programs (see Supplementary Table 5, Additional file 2). While a large number of breakpoints were shown across the length of the EHV2 genome, no significant recombination hot spots were detected.

## Discussion

Earlier studies have reported genetic diversity in EHV2 using different techniques, including restriction endonuclease digestion of viral DNA, antigenic studies and evolutionary studies [12, 14, 36, 54, 67]. Due to few available complete genome sequences for EHV2 and EHV5, partial and complete sequences of selected genomic regions such as gB, gH, and DNA polymerase have been analysed in various studies, and have consistently revealed a higher diversity for EHV2 compared with EHV5 [5, 83, 91]. The full genome sequences of eighteen EHV2 isolates generated in this study add substantially to the two full genome sequences previously reported [87, 102]. Genomic heterogeneity was observed between EHV2 viruses isolated from individual horses in this study supporting previous reports that genetically heterogenous strains of EHV2 can coinfect the same animal [7, 12, 14, 91].

Advances in high-throughput sequencing and genome-wide analyses of herpesviruses have aided the exploration of their diversity and evolution [69]. Analysis of the twenty EHV2 genome sequences revealed a high level of genomic diversity, consistent with previous reports of genetic diversity between EHV2 isolates [9, 12, 11, 14, 15, 75, 91]. The level of inter-strain nucleotide diversity of the EHV2 genomes is higher compared to the other herpesvirus genomes of EBV (gammaherpesvirus), EHV1 and EHV4 (alphaherpesviruses) and HCMV and murine cytomegalovirus (MCMV, betaherpesviruses) genomes reported elsewhere [64], [77], [79], [95].

Genetic variation is a putative driver of Kaposi’s sarcoma-associated herpesvirus (KSHV) and EBV infection, and may be associated with the site of isolation, clinical syndromes, and geographical location [18, 40, 63, 64, 84, 101]. Genomically distinct EHV2 viruses were isolated from individual horses in this and other studies [9, 11, 12, 14, 15, 75, 91]. Whether this variation is a driver of EHV2 infection remains to be determined, although high genetic diversity may enhance the ability of the virus to modulate host immunity [9, 25, 36, 37, 58, 62, 73, 91, 92].

Even though all the EHV2 isolates sequenced in this study were from Victoria, Australia, representatives of each genogroup of E1, E6 and ORF74 were found in this and other studies [75]. Similarly, variations in gB gene appeared to show no geographical association [5, 58, 83, 91]. These findings suggest there are no geographic associations with EHV2 genomic variation. This is in contrast to findings in some other gammaherpesviruses (EBV) where geographical association with genome sequence have been observed [17, 88, 103].

Variations in the nucleotide diversity of individual genes was observed throughout the length of EHV2 genome (Fig. 3b). This is consistent with reports in other herpesviruses including HCMV, human herpes simplex virus 1 (HSV1), EBV and KSHV, where isolates display uneven distribution of diversity, with high diversity observed in genes required for persistence viral infection and latency establishment, including structural genes and some glycoproteins [64, 70, 86, 101].

EHV2 genes that are more diverse have a range of roles in replication and pathogenesis. These include viral proteins that are targets of the immune response (gB, gH) [11, 36, 76, 82, 93] and some genes involved in viral immune evasion (ORFs 52 and 64, homologs of EBV BLRF2 and BPLF1) [24, 94], as well as in the establishment of latency (ORF73 homologue of KSHV latency associated nuclear antigen LANA-1) [31, 80, 100]. The diversity of these genes may be driven by pressure from escaping immune responses and establishing successful viral infection. Antigenic variation in neutralisation epitopes of EHV2 gB have been suggested as a means of immune escape and may drive some of the genetic diversity detected in gB and perhaps other glycoproteins [11, 36]. During the latent phase, maintenance of the episomal DNA requires replication and division of daughter cell nuclei, KSHV LANA-1 and EBV EBNA-1 are involved in tethering the viral episome to host cell chromatin to facilitate replication. This process entails modulating multiple cellular signalling pathway to recruit enzymes that modify chromatin, replication, and transcription factors to ensure persistent latent infection [6, 32, 68].

ORF51 is a unique gammaherpesvirus-gene with the homolog in EBV (BLLF1, gp350) known to mediate virus attachment to human B lymphocytes [94] and is a target of neutralizing antibodies in vivo [56, 90]. EHV2 ORF51 (second most diverse gene) in this study displays a higher diversity than EBV BLLF1, marked by a higher number of non-synonymous variations [78]. The function of ORF51 in EHV2 is unknown and prior to this study, this ORF was severely truncated in the first complete EHV2 sequence identified (86–67) [87] while the second complete genome G9/92 encodes the full gene [102]. This study found full length gp350 homologues are encoded in 16 of the 20 complete genomes now known. Future studies are required to elucidate whether this protein mediates attachment of EHV2 to B cells in a manner homologous to EBV, and how the high level of diversity relates to its function during infection.

Most EHV2-specific genes are highly diverse and are located near the genomic termini (E1-3, E6A, E9-10). This echoes findings from MCMV genome analyses where the level of genetic variability is highest at the genome termini and the most diverse genes are specific to MCMV [79]. Similarly, both ends of KSHV genome have hypervariable genes (K1 and K15) [74]. The presence of more lineage specific genes at the genomic termini is a consistent feature of herpesvirus evolution [20].

We identified 27 EHV2 genes with Ka/Ks ratios greater than 1 (Table S3) compared to only one such gene in the MCMV genome (m102.1) and no HCMV and HSV1 genes with Ka/Ks ratio > 1 [77, 79, 86]. Nucleotide diversity (π) and Ka/Ks ratio of EHV2 genes are not strongly correlated (Spearman *ρ* = 0.212). The Ka parameter has been suggested to be relatively consistent for defining gene divergence [46, 97]. Interestingly, some EHV2 genes, ORFs 70, 43, 65 and 39 (gM) with Ka values < 0.02 have very high Ka/Ks ratios (Ka = 0.01, 0.007, 0.15, 0.009,Ka/Ks = 6.5, 4.7, 4.6, 4.5 respectively) signifying diversifying selection, whereas gB (ORF8), gH (ORF22) and two of the 7TMR genes (ORFs 74 and E1) are highly divergent (Ka values > 0.025) but have Ka/Ks ratios < 0.5 indicating purifying selection (skewed by high levels of synonymous substitution [Ks]). Similarly, the MCMV gB, which is the target of most circulating anti-MCMV antibodies despite the variability it displays, has a Ka/Ks ratio of 0.18 which indicates strong purifying selection [79]. EBV latency associated genes (EBNA2, EBNA3 and LMP1) displayed the highest diversity of all EBV genes, marked by a greater extent of nonsynonymous variations (Ka = 0.06 compared to 0.086 in EHV2) [64]. Functional constraints on these genes may explain the selection pressure acting on them, and the variations could have been introduced through recombination [75]. These pressures may also exist for some of the conserved EHV2 genes, similar to what has also been reported in alphaherpesviruses [89]. The data and results from this study would be useful for future studies on the possible differences in prevalence, pathogenicity or tropism of different EHV2 variants.

Recombination is an important mechanism through which genetic differences that have arisen by mutation can be shuffled to further increase genetic variability and then re-distributed through viral populations [4]. This has been demonstrated for EBV latency associated genes which show high levels of diversity and have high recombination rates [8, 64, 101]. Previously, genetic variability of EHV2 genes such as gB, gH and the GPCRs (ORFs 74, E1 and E6) was suggested to be due to recombination [11, 75]. The number of recombination events detected in EHV2 (155 events in 20 genome sequences as detected by 3 or more programs, and 105 events as detected by 5 or more programs (see Supplementary Table 5, Additional file 2) is higher compared to EHV4 (5 events in 14 genome sequences as detected by 3 or more programs) [95], and MCMV (86 recombination events in 12 genome sequences as detected by 5 or more programs) [79] and consistent with recombination being a key driver of EHV2 genetic diversity [75]. Recombination and genomic variation are correlated in herpesviruses, including in the betaherpesvirus HCMV and several alphaherpesviruses such as HSV1, avian infectious laryngotracheitis virus (ILTV) and EHV4 [10, 50, 77, 95]. In all these herpesvirus species, high recombination rates have been attributed, in part, to a high prevalence of infection and high rates of co-infections in hosts. Infection with EHV2 is known to be highly prevalent in horse populations [30, 61, 83] and co-infection with different EHV2 strains is common in horses [9, 11, 12, 14, 75, 91]. In our study, we observed recombination breakpoints spread across the EHV2 genome, with more points concentrated in some genomic regions (genome termini) than in others. Similar observations have been made for other herpesviruses [64, 79]. Multiple reticulate network trees revealed extensive recombination amongst EHV2 isolates as shown by isolates clustering in different groups when different genome regions or individual genes were analysed (complete, unique, and repeat regions, or gB, gH, and the GPCRs). In addition, EHV2 isolates from the same horse did not share the same genotype group for all the three GPCR genes, the gB gene and the gH gene.

## Conclusion

Our understanding of the genome diversity of EHV2 increases with the availability of sequence data. The 18 EHV2 full-genome sequences generated from this study contribute to genomic studies of EHV2. Analyses of the resultant 20 genome sequences enabled us to assess EHV2 genetic diversity and recombination in genomic regions and individual genes. Our findings point to notable or unique characteristics of EHV2 compared to many other herpesvirus species, including a comparatively high number of recombination breakpoints, a high level of genetic diversity and a large proportion of genes seemingly undergoing diversifying selection. These results are likely to reflect the biology of this evolutionarily successful virus, including its infection and immune evasion characteristics and the high prevalence of infection in horses, as well as co-infections in individual hosts.

## Methods

### Viruses and cells

Eighteen Australian isolates of EHV2 were used in this study, including ten archived viruses isolated between 1967 and 1994 and eight isolates collected for this study in 2018 (Table 2).

To isolate the contemporary viruses, PBMCs were isolated from two ponies and three Thoroughbred (TB) horses in Victoria, Australia. Whole blood was collected with approval from the Faculty of Veterinary and Agricultural Sciences Animal Ethics Committee at the University of Melbourne (approval number 1714237.1). Whole blood was collected from the 5 horses (80 ml of blood per horse was collected into heparin treated tubes, final concentration 20 IU/ml) and immediately chilled on ice and transported to our laboratories for Ficoll-Paque purification as previously described [55]. Approximately 10^6^ PBMCs in 500 µl volumes were overlayed onto primary equine foetal kidney (EFK) cell monolayers under methyl cellulose overlay media in 6-well trays [85]. Isolated viral plaques were picked and identified as either EHV2 or EHV5 by PCR screening [27] before selected plaques were plaque-purified on EFK monolayers two more times followed by amplification of virus stocks and virus purification using sucrose or Ficoll gradients as previously described [2, 85]. PCR identification of these plaques is summarised in Table 1.

To select a range of distinct viruses for sequencing from the contemporary collection, qPCR-HRM curve analysis of a region of EHV2 gB was used to compare EHV2 isolates. Primers corresponding to nucleotides (nt) 2096—2115 (forward primer 5*'* -GCAAGGTGGTGGTCAATGTG -3*'*) and 2535—2555 (reverse primer 5*'* GGCTCATAATCCCCCTCATCG -3*'*) were used, numbered according to the EHV2.86–67 genome (GenBank accession NC_001650). Details of the isolates selected for whole genome sequencing are shown in Tables 1 and 2.

### DNA extraction, genome sequencing and assembly

Viral DNA was extracted from purified virus using the High Pure PCR template preparation kit (Roche) according to the manufacturer’s instructions. Following DNA extraction, sequencing of all the selected isolates was performed as described previously [50]. Briefly, libraries were prepared using 1 ng of DNA with the Illlumina Nextera XT kit and sequenced according to the manufacturer’s instruction using Illumina MiSeq with V3 chemistry (Illumina 15,046,563 v02). The reads produced consist of paired end read of 150 bp.

All the analysis performed in Geneious was done using version 11.1.5 (www.geneious.com).

The quality of the resulting paired end sequencing reads was assessed using FASTQC software (https://www.bioinformatics.babraham.ac.uk/projects/fastqc/) [3]. Read quality was processed using BBDuk 1.0, a plugin in Geneious [43]. Assembly was done using Geneious mapper at medium–low sensitivity option with 5 iterations as recommended by the program [95]. Sequence reads were assembled by first mapping to the reference strain EHV2 86–67 (NC_001650) excluding the first terminal repeat region, with a modification of the maximum mismatch allowance to 40% to generate a ‘preliminary genome sequence’ for each isolate. The ‘generate consensus sequence’ option in Geneious was used to derive the consensus sequences based on the majority of nucleotides in the coverage area at a cut off of 30. All other parameters used default settings. The genome assembly of each isolate was completed by iteratively mapping the sequence reads of each isolate onto its preliminary genome sequence until the entire genome length was covered, or the number of reads mapped to the preliminary genome sequence stopped increasing. The reference sequence was used in areas with low coverage and quality.

De novo assembly was used to assemble two of the genomes (Fin60-72 and 157IFEye-69) to compare the two methods of genome assembly as described previously [95]. The alignment of the two genome sequences derived from the two methods showed some differences in selected genome regions, particularly in repeat rich areas. The sequence reads were remapped separately to each genome sequence as reference to closely examine the regions of disagreement, which are shown to be mostly associated with areas containing repeats and INDELS. Some CDS regions showing disagreement, specifically those with large INDELs, were characterized using PCR amplification and analysis of amplicon size using the following primes (ORFs 29: F—TCAGGGTGTTGGAGTTGAGC, R – TACACCAACAACACGGAGGC,34: F—CTTGCAGTACGAGTCCAGCA, R – AACGCCTCAGAGAACCGC; and 48: F—GATTTCTTTCTTCGCCCCCG, R – CATCTCTGGGGAAGTTGGCC).

### Genome annotation and sequence alignment

The annotations from the reference genome (86/67) were transferred to the new genome sequences using the “Annotate From” and “Transfer Annotation” functions in Geneious on default settings. Open reading frames (ORFs) were detected by using Geneious ‘Find ORFs’ option. ORFs were recognized as initiated by the start codon (ATG), ending with a termination codon (TGA, TAA or TAG) and a minimum length of 120 bp. The transferred annotations were then curated using the predicted ORF sizes. ORFs were individually extracted and genomic features in EHV2 strains 86–67 and G9/92 were verified by BLAST searches of the GenBank database (National Center for Biotechnology Information (NCBI) website http://www.ncbi.nlm.nih.gov/BLAST/ using BLASTn, BLASTx, tBLASTx.

The complete genome sequences of all 18 EHV2 isolates derived from this study were aligned together with strains 86–67 and G9/92 using the Multiple Alignment with Fast Fourier Transformation (MAFFT) 7.450 plugin in Geneious [41, 42]. The prototype strain EHV2 86–67 [87] as used as reference sequence for the alignments. To extend the analysis of some EHV2 genes, such as glycoproteins and GPCRs beyond 18 sequences generated in this study, publicly available complete sequences of these genes in NCBI database were included in our analyses (see Supplementary Table 4, Additional file 2). Comparative analysis of aligned complete genome and selected genes sequences were performed in Geneious 11.1.5.

### Comparative sequence analysis

To perform phylogenetic analysis, the best-fit model was first determined using the IQ-TREE web server http://iqtree.cibiv.univie.ac.at/ [59]. GTR model was indicated as best fit with a gamma distribution and BIONJ tree builder. The maximum likelihood phylogenetic analysis was performed on the alignments of complete EHV2 gB, gH, E1, E6 and ORF74 genes from this study and those sourced from NCBI (see Supplementary Table 4, Additional file 2), and complete genome (excluding 1TR) using GTR model (GTR + 1 + G4) within the Molecular Evolutionary Genetics Analysis (MEGA) version 7 [47, 96]. Trees were initially built using an improved version of Neighbor joining (NJ) algorithm (BIONJ) and Nearest-Neighbor-Interchange (NNI) for Heuristic analysis using the Maximum Composite Likelihood (MCL) with 1000 bootstrap replicates and a support threshold of 90% [33].

The DNA polymorphism option of the DNA Sequence Polymorphism (DnaSP) software 6.12.01 × 64 [72] was used to calculate whole genome diversity parameters, including nucleotide diversity (π), the number of polymorphic sites, and the average number of nucleotide differences, excluding gapped sites. Window size and step size were set at 500 nt and 100 nt, respectively, as previously described [77]. Also, nonsynonymous substitutions per nonsynonymous site (Ka) and synonymous substitutions per synonymous site (Ks) were calculated using the synonymous and nonsynonymous substitutions option [72].

Sequence similarity plots between EHV2 strains were performed using SimPlot software version 3.5.1 as previously described [49]. This software calculates and plots the percent identity of the query sequence to a panel of reference sequences in a sliding window, which is moved across the alignment in steps.

### Recombination analysis

In preparing the aligned genome sequences for recombination analysis, tandem repeat regions were first identified and deleted using Phobos [53] plugin in Geneious as previously described [95]. Recombination analysis was performed on aligned complete genome sequences, individual unique region, internal repeats (IR1 and IR2) and terminal repeat regions. Evidence of intraspecific recombination was examined using recombination detection programs, RDP4 version 4.95 [51, 52] and SplitsTree4 V 4.14.6 [39]. Recombination network trees were generated as previously described [95] and the pair-wise homoplasy (Phi) test [15] was used to analyse the statistical significance of recombination networks as executed within SplitsTree4. Six different programs in RDP4 were executed using default setting to detect recombination breakpoints including RDP, GENECONV, 3Seq, Maximum Chi Square (MaxChi), SiScan and BootScan [51, 52]. Only break points detected by at least three programs with a Bonferroni-corrected *P* value < 0.05 were reported. Duplicate and flagged breakpoints were omitted. A plot of the distribution of recombination break points along the length of the genome was generated as previously described [50].

## Supplementary Information

**Additional file 1:** **Figure S1. **Comparison of the genome sequence assembly method of EHV2 157IFEye-69. Genome sequence as determined by *de novo* assembly (157IFEye-69 *de novo*) and by mapping against the reference strain sequence EHV2 86/67 (GenBankaccession number NC_001650) (157IFEye-69 map to ref). Vertical black lines indicate single nucleotide polymorphism differences between the genome assemblies and dashes indicate sequence gaps. **Figure S2.** Comparison of the genome sequence assembly of EHV2 Fin60-72. Genome sequence as determined by *de novo* assembly (Fin60-72 *de novo*) and by mapping against the reference strain sequence EHV2 86/67 (GenBank accession number NC_001650) (Fin60-72 map to ref). Vertical black lines indicate differences between the genomes and dashes indicate sequence gaps. **Figure S3.** Whole-genome alignments and similarity plots of two EHV2 isolates (18-2018 and 147-2018) recovered from Horse 4. Alignments of genome sequences (excluding 1 terminal repeat) of the isolates. Each point plotted is the percent identity within a sliding window of 10,000 bp wide centered on the position plotted, with a step size of 20 bp. Vertical black lines indicate single nucleotide differences between the isolates and dashes indicate sequence gaps. Results indicate varying degrees of genetic heterogeneity at various genome regions between the EHV2 strains within an individual horse. **Figure S4.** Whole-genome alignments and similarity plots of three EHV2 isolates (60-2018, 91-2018, and 57-2018) recovered from Horse 5. Alignments of genome sequences (excluding 1 terminal repeat) of the isolates. Each point plotted is the percent identity within a sliding window of 10,000 bp wide centered on the position plotted, with a step size of 20 bp. Vertical black lines indicate single nucleotide differences between the isolates and dashes indicate sequence gaps. Results indicate varying degrees of genetic heterogeneity at various genome regions between the EHV2 strains within an individual horse. **Figure S5.** Recombination network trees for 5 complete EHV2 genes (nucleotide sequences) (a) gB, (b) gH, (c) E6, (d) E1 and (e) ORF74 from a panel of EHV2 field isolates. The Phi test for detecting recombination as implemented in SplitsTree4 was significant for all the genes *P* < 0.05. The multiple reticulate networks indicate recombination between the different isolates as previously proposed (Sharp et al. 2007). Isolates in oval (red or black) indicate those recovered from the same horse. The bar indicates the rate of evolution in sequence substitutions per site.

**Additional file 2:** **Table S1.** Summary of sequencing metrics and assembly for EHV2 isolates. **Table S2.** Percentage nucleotide identity between EHV2 isolates. **Table S3.** Analysis of the overall selective pressure acting on EHV2 genes. Calculations of non-synonymous substitutions per non-synonymous site (Ka) to synonymous substitution per synonymous site (Ks) ratios for all EHV2 genes, along with standard errors based on 1,000 bootstrap replicates. **Table S4.** Details of the genes included in the study and their accession numbers. **Table S5.** Recombination breakpoint analysis of 20 EHV2 strains including strains 86/67 and G9/92.

## Data Availability

The datasets generated and /or analysed during the current study are available in the GenBank repository under accession numbers MW322569 to MW322586 (Table 2 and Supplementary Table 4, Additional file 2). The sequence reads have been deposited in the Sequence Read Archive (SRA) https://www.ncbi.nlm.nih.gov/sra/PRJNA797706 under accession numbers SRR17631872 to SRR17631889 as listed in Table 2.
