# Supplementary figures and images for: Whole genome sequence analysis of equid gammaherpesvirus -2 field isolates reveals high levels of genomic diversity and recombination
Source: BMC Genomics. 2022 Aug 30;23:622. doi: 10.1186/s12864-022-08789-x (PMC9426266; doi:10.1186/s12864-022-08789-x)

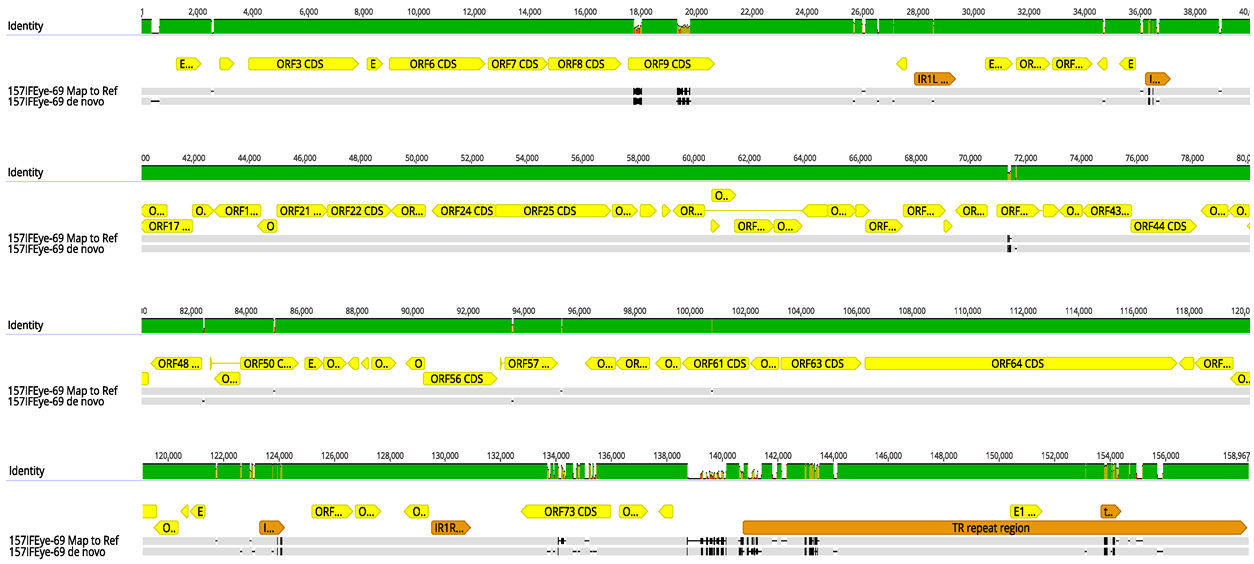


**
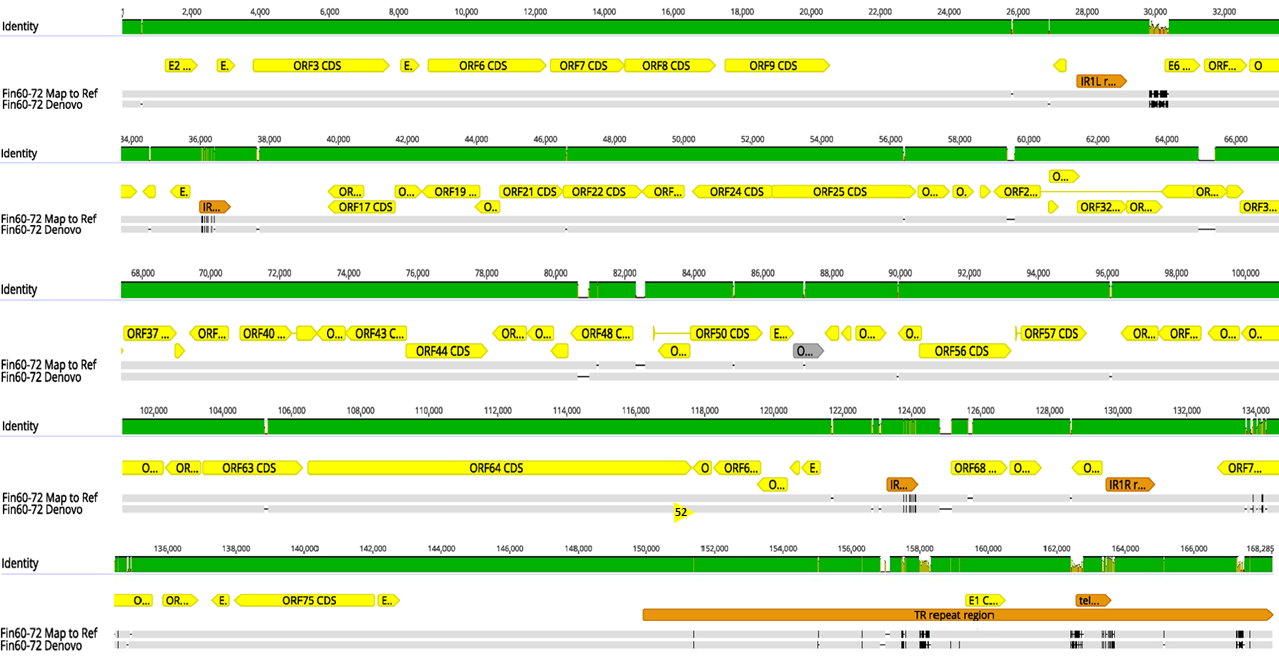
**

**
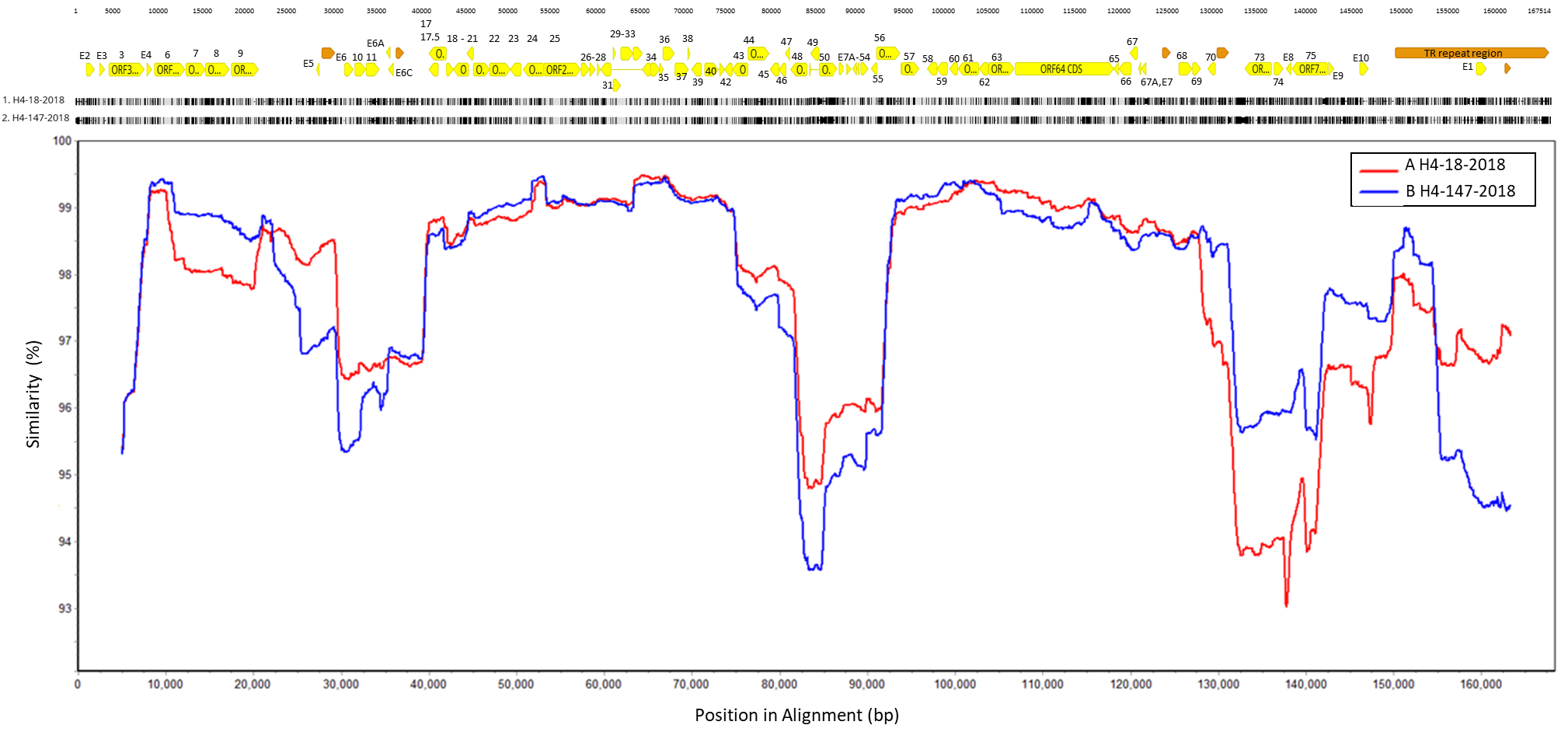
**

**
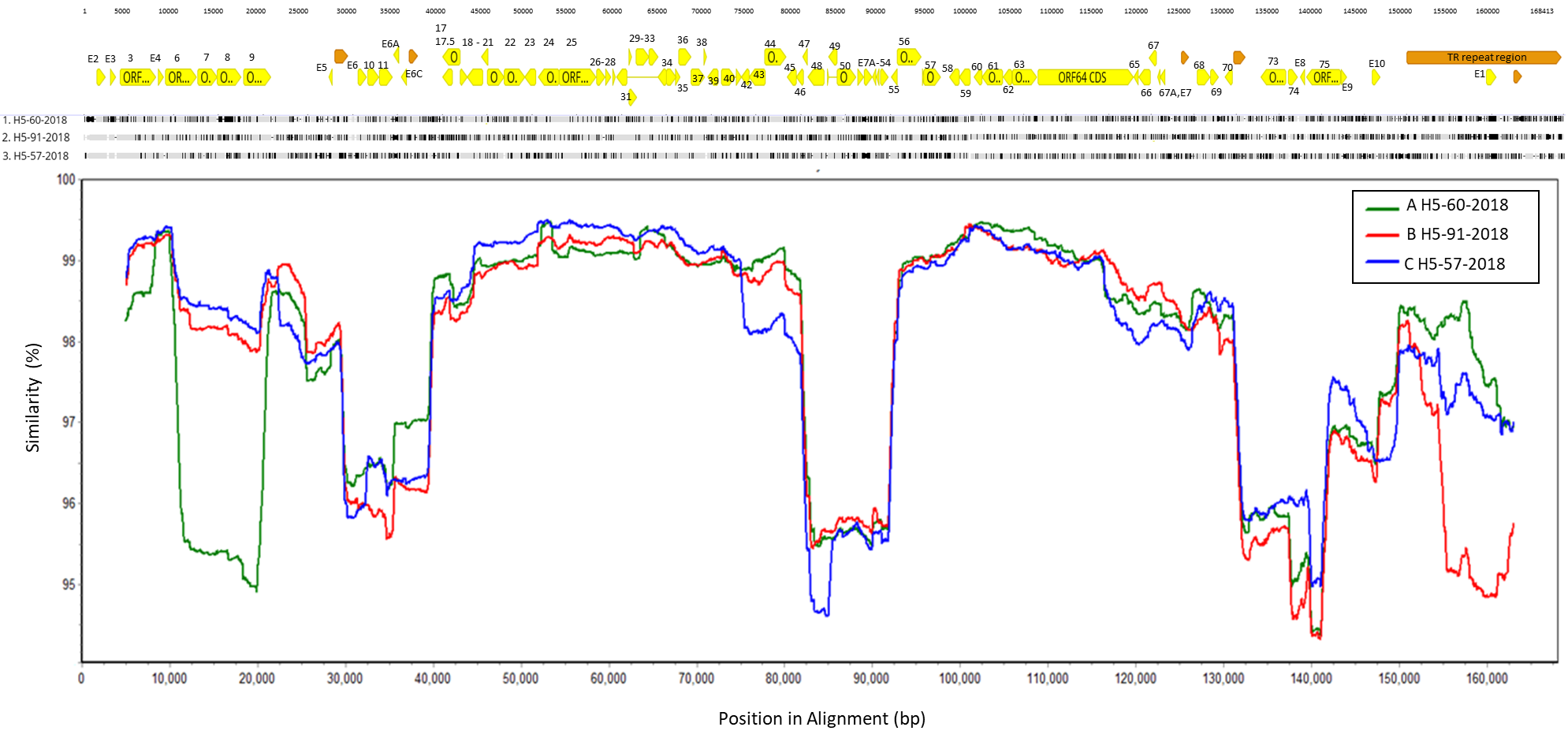
**

**
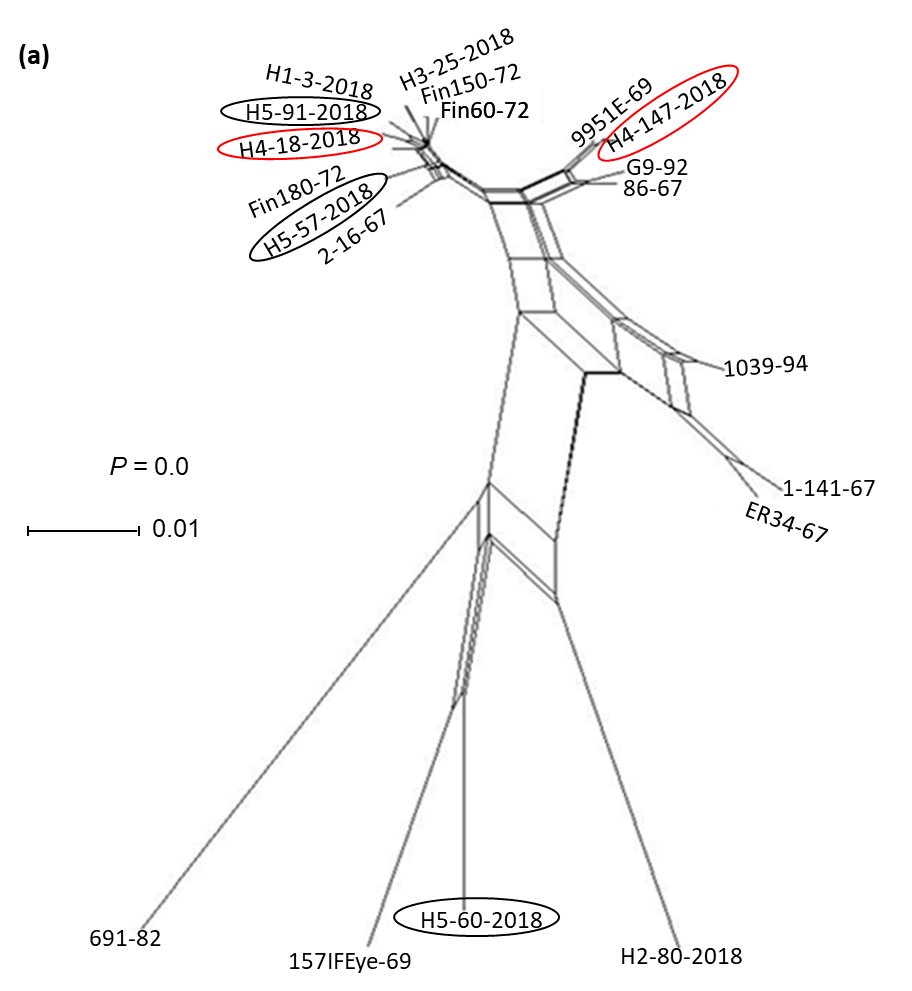
**

**
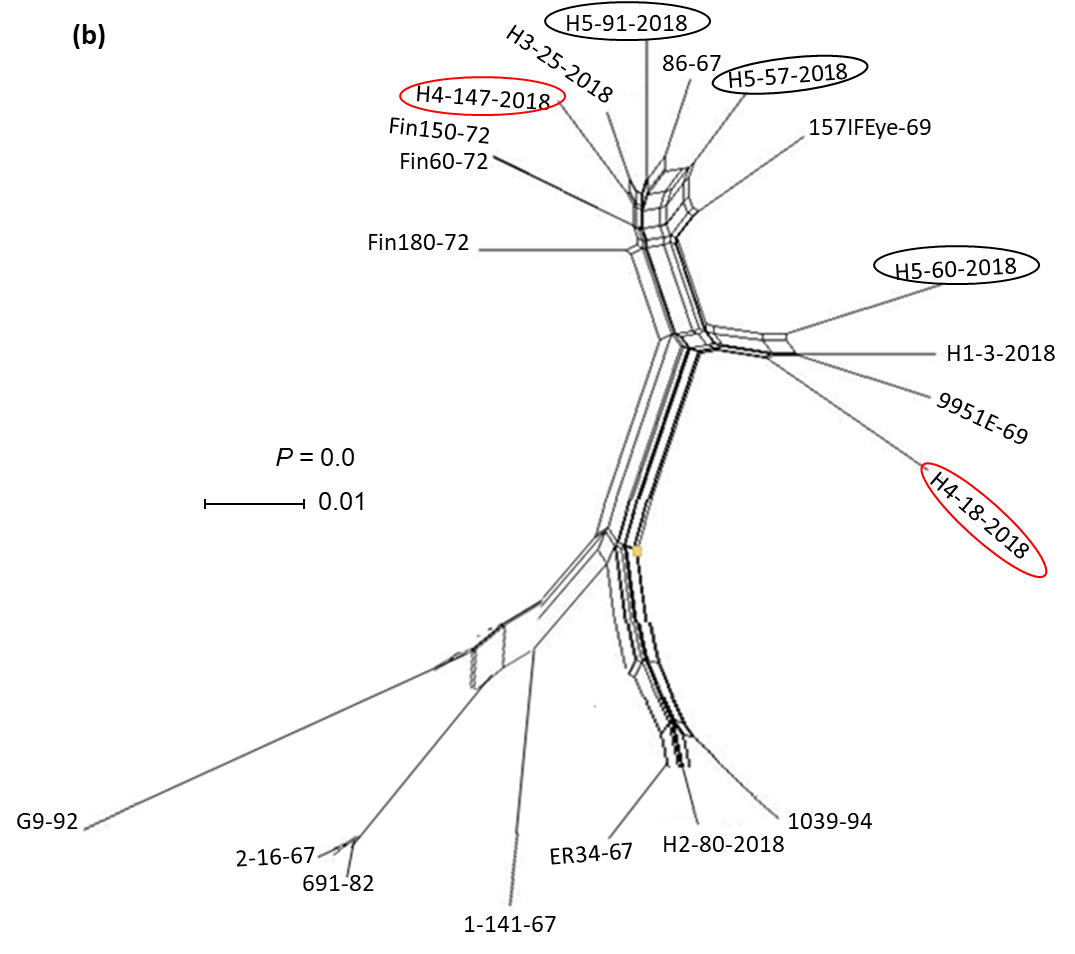
**

**
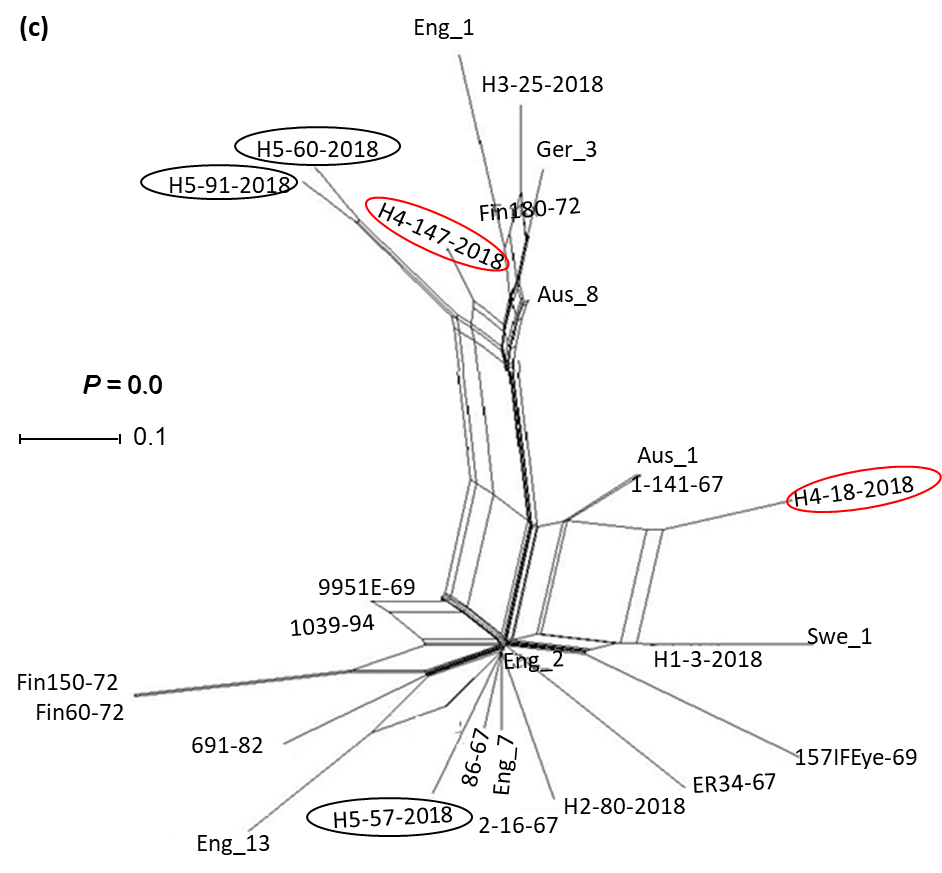
**

**
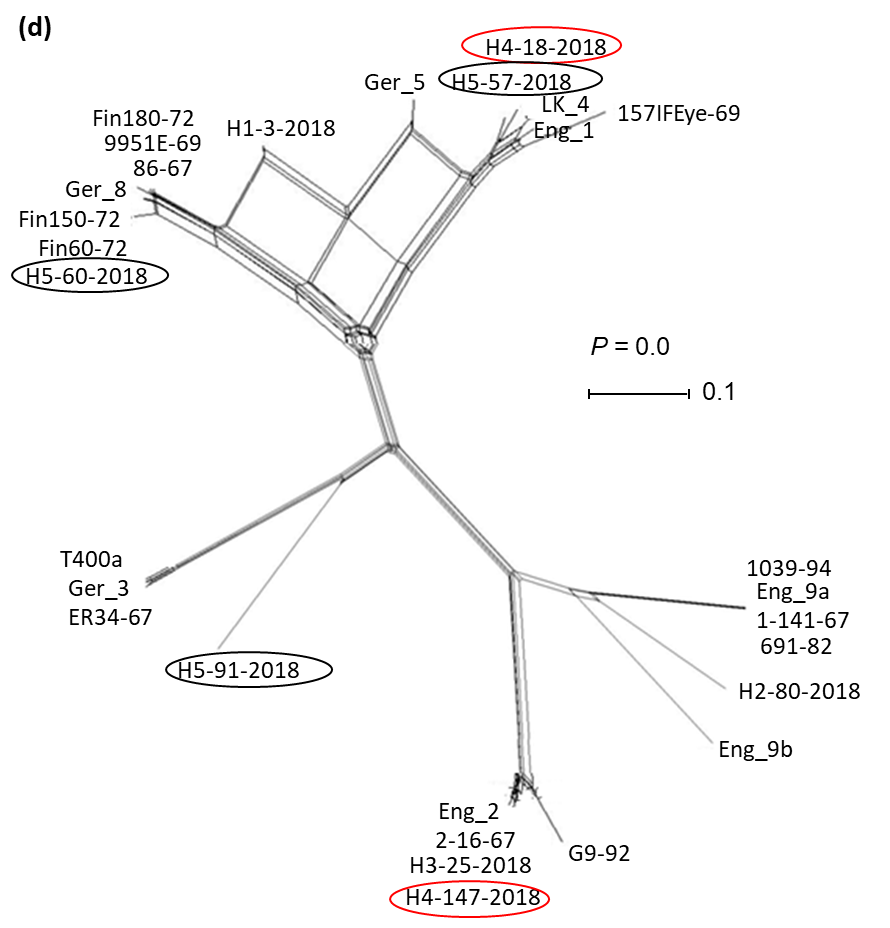
**

**
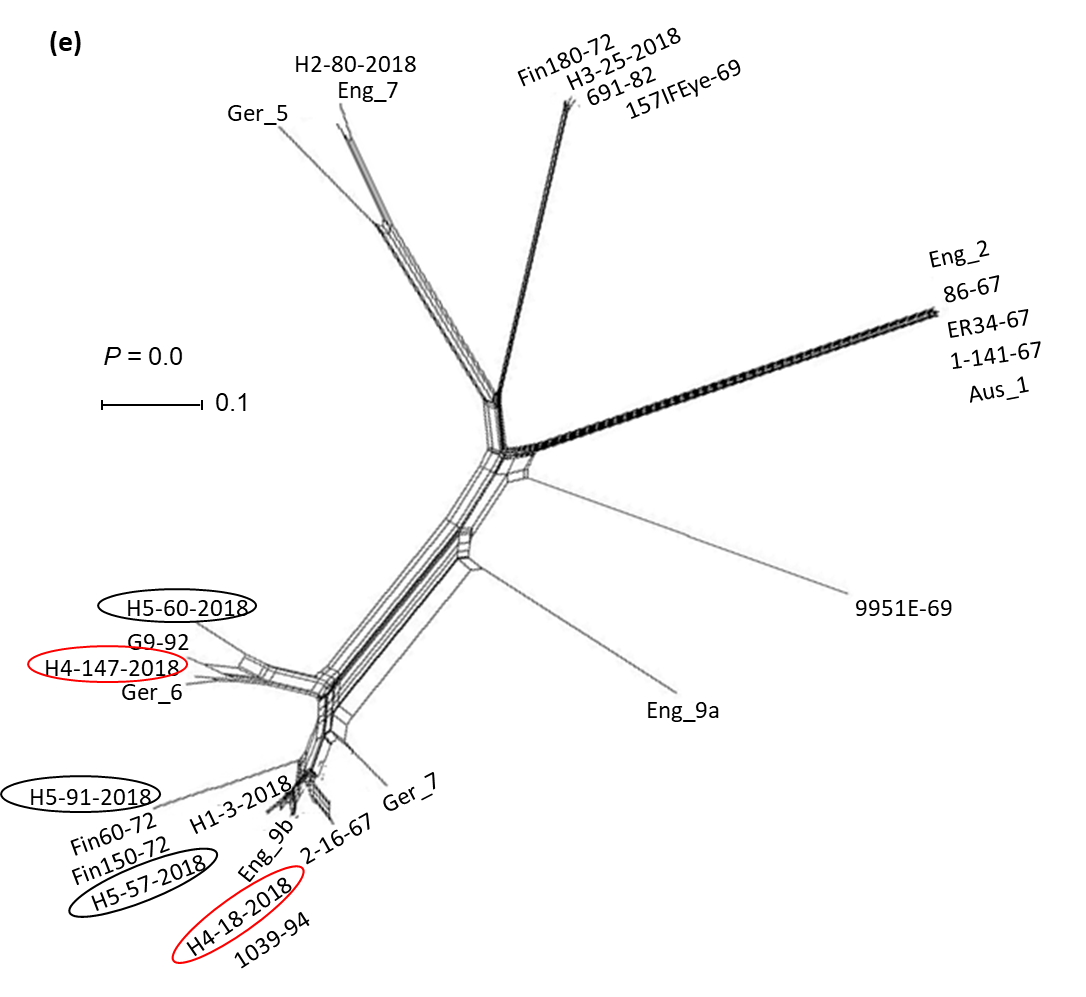
**

Supplement: Supplementary file 1 — Additional file 1: Figure S1. Comparison of the genome sequence assembly method of EHV2 157IFEye-69. Genome sequence as determined by de novo assembly (157IFEye-69 de novo) and by mapping against the reference strain sequence EHV2 86/67 (GenBankaccession number NC_001650) (157IFEye-69 map to ref). Vertical black lines indicate single nucleotide polymorphism differences between the genome assemblies and dashes indicate sequence gaps. Figure S2. Comparison of the genome sequence assembly of EHV2 Fin60-72. Genome sequence as determined by de novo assembly (Fin60-72 de novo) and by mapping against the reference strain sequence EHV2 86/67 (GenBank accession number NC_001650) (Fin60-72 map to ref). Vertical black lines indicate differences between the genomes and dashes indicate sequence gaps. Figure S3. Whole-genome alignments and similarity plots of two EHV2 isolates (18-2018 and 147-2018) recovered from Horse 4. Alignments of genome sequences (excluding 1 terminal repeat) of the isolates. Each point plotted is the percent identity within a sliding window of 10,000 bp wide centered on the position plotted, with a step size of 20 bp. Vertical black lines indicate single nucleotide differences between the isolates and dashes indicate sequence gaps. Results indicate varying degrees of genetic heterogeneity at various genome regions between the EHV2 strains within an individual horse. Figure S4. Whole-genome alignments and similarity plots of three EHV2 isolates (60-2018, 91-2018, and 57-2018) recovered from Horse 5. Alignments of genome sequences (excluding 1 terminal repeat) of the isolates. Each point plotted is the percent identity within a sliding window of 10,000 bp wide centered on the position plotted, with a step size of 20 bp. Vertical black lines indicate single nucleotide differences between the isolates and dashes indicate sequence gaps. Results indicate varying degrees of genetic heterogeneity at various genome regions between the EHV2 strains within an indivi [file 12864_2022_8789_MOESM1_ESM.docx]
